# Supplementary material for: Improving classification of correct and incorrect protein–protein docking models by augmenting the training set
Source: Bioinform Adv. 2023 Feb 2;3(1):vbad012. doi: 10.1093/bioadv/vbad012 (PMC9923443; doi:10.1093/bioadv/vbad012)
Supplement: vbad012_Supplementary_Data [file vbad012_supplementary_data.pdf]

Table S1 . The Capri quality definitions, depend on different combinations of LRMSD, IRMSD and fnat. For most of the categories there is a need to fulfill one of the two conditions indicated on each columns.

Quality	Condition 1	Condition 2
Incorrect	LRMSD > 10 Åand IRMSD > 4 Å fnat < 0.1	fnat < 0.1
Acceptable	(0.1 < fnat < 0.3) and (LRMSD ≤ 10 Å or IRMSD ≤ 4 Å)	fnat ≤ 0.3 and (LRMSD > 5 Å or IRMSD > 2 Å)
Medium	0.3 ≤ fnat < 0.5) and (LRMSD ≤ 5.0 Å or IRMSD ≤ 2 Å	fnat ≥ 0.5 and (LRMSD > 1.0 Å and IRMSD > 1.0 Å)
High	fnat ≥ 0.5 and (LRMSD ≤ 1 Å or IRMSD ≤ 1 Å)	

Table S2 List of selected features used for the analysis and training. For each feature, the public source, relative reference and a short description are provided.

Feature	Source	Description	Reference
**CONSRANK_val**	CONSRANK	CONSRANK normalized score, reflecting the conservation of the inter- residue contacts of a given model in the decoys set	Bioinformatics 31:1481 (2015)
**AP_GOAP_DF**	CCharPPI	The DFIRE term in the GOAP energy	Biophys J. 2011 101(8): 2043-2052.
**CP_MJ3h**	CCharPPI	Contact potential, calculated between intermolecular residues	Proteins 59(1):49 (2005) and BMC Bioinformatics 11:92 (2010)
**DDG_V**	CCharPPI	A microscopic surface energy model derived from mutation data	Proteins 83(4):640 (2015).
**CP_HLPL**	CCharPPI	Contact potential, calculated between intermolecular residues	Proteins 59(1):49 (2005) and BMC Bioinformatics 11:92 (2010)

**CP_RMFCA**	CCharPPI	The C_alpha-C_alpha potential	Proteins 65(3):726 (2006).
**CP_TD**	CCharPPI	Contact potential, calculated between intermolecular residues	Proteins 59(1):49 (2005) and BMC Bioinformatics 11:92 (2010)
**CP_SKOIP**	CCharPPI	The residue level interaction contact potential	Biophys. J. 84(3):1895 (2003).
**CP_D1**	CCharPPI	A reimplementation of the DECK residue level distance-dependent potential	BMC Bioinformatics. 2011 12:280.
**CP_Qp**	CCharPPI	Contact potential, calculated between intermolecular residues	Proteins 59(1):49 (2005) and BMC Bioinformatics 11:92 (2010)
**SIPPER**	CCharPPI	The SIPPER potential, described in J, Chem, Inf, Mod, 51	J. Chem. Inf. Mod. 51:370 (2011).
**AP_DFIRE2**	CCharPPI	Interaction energy calculated using the DFIRE2 potential	Protein Sci. 17:1212 (2008).
**AP_dDFIRE**	CCharPPI	Interaction energy calculated using the dDFIRE potential	Proteins 72:793 (2008).
**PYDOCK_TOT**	CCharPPI	Total pyDock energy	Proteins 68:503 (2007) and Protein 69:852 (2007).
**CP_RMFCEN1**	CCharPPI	The 6bin-HRSC centroid-centroid potential	Proteins 70(3):950 (2006).
**CP_RMFCEN2**	CCharPPI	The 7bin-HRSC centroid-centroid potential	Proteins 70(3):950 (2006).
**CP_TSC**	CCharPPI	The residue level interaction two-step potentia	BMC Struct biol 10:40 (2010).
**CP_MJ2h**	CCharPPI	Contact potential, calculated between intermolecular residues	Proteins 59(1):49 (2005) and BMC Bioinformatics 11:92 (2010)
**CP_TB**	CCharPPI	The residue level interaction contact potential	Proteins 62(4):970 (2006).
**BSA_Apolar**	Freesasa	Polar Buried Surface Area from FreeSASA	F1000Research 5:189 (2016)

**CP_BT**	CCharPPI	Contact potential, calculated between intermolecular residues	Proteins 59(1):49 (2005) and BMC Bioinformatics 11:92 (2010)
**AP_DDG_W**	CCharPPI	The weighted atomic potential derived from mutation data	J. Chem. Theory Comput. 9(8):3715-3727 (2013).
**AP_DARS**	CCharPPI	The DARS potential	Biophys J. 2008 95(9):4217-27
**FIREDOCK**	CCharPPI	The total FireDock energy (default energy function)	Proteins 69(1):139 (2007).
**AP_PISA**	CCharPPI	The PISA score	Proteins 81(4):592 (2013).
**CP_TEl**	CCharPPI	Contact potential, calculated between intermolecular residues	Proteins 59(1):49 (2005) and BMC Bioinformatics 11:92 (2010)
**CP_TS**	CCharPPI	Contact potential, calculated between intermolecular residues	Proteins 59(1):49 (2005) and BMC Bioinformatics 11:92 (2010)
**AP_DDG_U**	CCharPPI	The unweighted atomic potential derived from mutation data	J. Chem. Theory Comput. 9(8):3715-3727 (2013).
**CP_ZS3DC_MIN**	CCharPPI	The E_ZS3DC z-score R_min potential	Protein Sci. 2011 20(3):529-41.
**CP_BFKV**	CCharPPI	Contact potential, calculated between intermolecular residues	Proteins 59(1):49 (2005) and BMC Bioinformatics 11:92 (2010)
**PROPNSTS**	CCharPPI	Amino acid propensity score	J. Chem. Inf. Mod. 51:370 (2011).
**cips_AlAr**	CIPS	Sum of CIPS score for Aliphatic-Aromatic contacts	Bioinformatics 34:459 (2018)
**AlAr**	COCOMAPS	Aliphatic-Aromatic contact count at 5 Å distance	Bioinformatics 27:2915 (2011)
**CP_MJPL**	CCharPPI	Contact potential, calculated between intermolecular residues	Proteins 59(1):49 (2005) and BMC Bioinformatics 11:92 (2010)

**CP_SKOb**	CCharPPI	Contact potential, calculated between intermolecular residues	Proteins 59(1):49 (2005) and BMC Bioinformatics 11:92 (2010)
**AP_MPS**	CCharPPI	The MPS potential	Biophys J. 2008 95(9):4217-27
**ArAr**	COCOMAPS	Aromatic-Aromatic contact count at 5 Å distance	Bioinformatics 27:2915 (2011)
**RFC**	This work	Random forest classifier	
**NNC**	This work	Perceptron classifer	
**TF**	This work	Deep neural network classifer	

Table S3. Summary report from snorkel summary report for the labelling functions. We generated labeling functions using the top 30 scoring functions which had varying accuracies ranging from 66% to 84% (Initial optimization). For each scoring function, we aimed to find the thresholds that maximize the true positive rate (TPR) and the true negative rate (TNR). Optimizing snorkel using this approach resulted in modest results, achieving about 74% labeling accuracy on the training dataset and about 70% labeling accuracy on the validation dataset. The results had a small variance when training snorkel with either top 10, top 20, or top 30 scoring functions. The final set of labelling functions (Final optimization) presented better accuracy on the train and testing set (86.26% and 87.71% respectively) at the cost of reducing the coverage but avoiding the high overlap presented on the initial optimization.

Initial optimization	j	Polarity	Coverage	Overlaps	Conflicts	Correct	Incorrect	Emp. Acc.
lf_CONSRANK_val	0	[0, 1]	0.999982	0.999982	0.924374	47025	8722	0.843543
lf_CP_HLPL	1	[0, 1]	0.995516	0.995516	0.919907	39622	15876	0.713936
lf_CP_MJ3h	2	[0, 1]	0.998726	0.998726	0.923118	39677	16000	0.712628
lf_DDG_V	3	[0, 1]	0.999982	0.999982	0.924374	39669	16078	0.711590
lf_CP_RMFCA	4	[0, 1]	0.999982	0.999982	0.924374	39463	16284	0.707895
lf_AP_GOAP_DF	5	[0, 1]	0.999982	0.999982	0.924374	39757	15990	0.713168
lf_CP_Qp	6	[0, 1]	0.993973	0.993973	0.918437	38619	16793	0.696943
lf_CP_TD	7	[0, 1]	0.999193	0.999193	0.923603	39688	16015	0.712493
lf_CP_SKOIP	8	[0, 1]	0.998099	0.998099	0.922490	39032	16610	0.701484
lf_CP_TB	9	[0, 1]	0.999856	0.999856	0.924248	38197	17543	0.685271
lf_CP_TSC	10	[0, 1]	0.999677	0.999677	0.924087	38290	17440	0.687063
lf_PYDOCK_TOT	11	[0, 1]	0.999982	0.999982	0.924374	38090	17657	0.683265
lf_SIPPER	12	[0, 1]	0.999928	0.999928	0.924320	38705	17039	0.694335

lf_CP_BT	13	[0, 1]	0.998404	0.998404	0.922795	37567	18092	0.674949
lf_CP_MJ2h	14	[0, 1]	0.999856	0.999856	0.924248	38440	17300	0.689630
lf_AP_DFIRE2	15	[0, 1]	0.999982	0.999982	0.924374	38833	16914	0.696594
lf_CP_RMFCEN1	16	[0, 1]	0.999767	0.999767	0.924159	38617	17118	0.692868
lf_AP_DARS	17	[0, 1]	1.000000	1.000000	0.924392	37980	17768	0.681280
lf_AP_PISA	18	[0, 1]	1.000000	1.000000	0.924392	37548	18200	0.673531
lf_BSA_Apolar	19	[0, 1]	1.000000	1.000000	0.924392	38100	17648	0.683433
lf_CP_BFKV	20	[0, 1]	0.999964	0.999964	0.924356	37245	18501	0.668120
lf_AP_dDFIRE	21	[0, 1]	0.999265	0.999265	0.923674	38233	17474	0.686323
lf_CP_RMFCEN2	22	[0, 1]	0.999785	0.999785	0.924177	38274	17462	0.686702
lf_CP_ZS3DC_MIN	23	[0, 1]	1.000000	1.000000	0.924392	36264	19484	0.650499
lf_AP_DDG_U	24	[0, 1]	0.999982	0.999982	0.924374	36939	18808	0.662619
lf_AP_DDG_W	25	[0, 1]	0.999982	0.999982	0.924392	37698	18049	0.676234
lf_cips_AlAr	26	[0, 1]	1.000000	1.000000	0.924392	36932	18816	0.662481
lf_CP_MJPL	27	[0, 1]	0.998888	0.998888	0.923334	37111	18575	0.666433
lf_CP_SKOb	28	[0, 1]	0.987856	0.987856	0.912553	36336	18735	0.659803
lf_CP_TEl	29	[0, 1]	0.999354	0.999354	0.923764	37411	18301	0.671507

Final optimization	j	Polarity	Coverage	Overlaps	Conflicts	Correct	Incorrect	Emp. Acc.
lf_CONSRANK_val	0	[0, 1]	0.218232	0.174141	0.010494	11898	268	0.977971
lf_CP_HLPL	1	[0, 1]	0.244583	0.210106	0.005058	11659	1976	0.855079
lf_CP_MJ3h	2	[0, 1]	0.231937	0.189011	0.008825	11166	1764	0.863573
lf_DDG_V	3	[0, 1]	0.238197	0.210716	0.007857	11351	1928	0.854808
lf_CP_RMFCA	4	[0, 1]	0.236690	0.211685	0.006224	11192	2003	0.848200
lf_AP_GOAP_DF	5	[0, 1]	0.226645	0.198805	0.009041	11157	1478	0.883023
lf_NNC	6	[0, 1]	0.211254	0.197047	0.004377	11723	54	0.995415
lf_TF	7	[0, 1]	0.215165	0.210788	0.003946	11914	81	0.993247

Supplementary Section 4. Classifiers details and hyperparameter

Perceptron

The hyperparameters for the perceptron consisted on the learning rate (alpha) of 3.7e-06 ,a batch size of 512 samples , and one hidden layer of 24 neurons.

Random Forest Classifier

This classifiers parameters consisting on 100 classifiers to make the forest, a minimum number of samples to split the node of ten, and weighted classes (Incorrect: 1.2 and correct : 0.1).

Deep neural network

We trained a sequential deep neural network using Tensorflow 2.0 framework and keras. We combined dense and dropout layers, the latter to drop 20% of the neurons. This model has a total of 2,665 trainable parameters.

Type	Output Shape	Param num
Dense	(None, 40)	360
Dense	(None, 16)	656
Dropout	(None, 16)	0
Dense	(None, 16)	272
Dense	(None, 16)	272
Dropout	(None, 16)	0
Dense	(None, 16)	272
Dense	(None, 16)	272
Dropout	(None, 16)	0
Dense	(None, 16)	272
Dense	(None, 16)	272
Dense	(None, 1)	17

Table S5. Success rate on the **3K-BM5up** for all the 20 best performing classifiers/scoring functions in terms of percentage of targets featuring at least one correct solution within the top-10 ranked positions. Values for the top-1 and top-100 positions are also reported.

**3K-BM5up**			
**Method**	**Top-1**	**Top-10**	**Top-100**
**RF-hAIkal**	50.0	55.8	69.2
**PRC-hAIkal**	43.6	54.6	69.1
**RF-core**	32.7	54.6	69.1
**PRC-core**	43.6	50.9	56.4
CP_TSC	14.6	49.1	65.4
**TF-core**	30.9	43.6	65.4
**TF-hAIkal**	29.1	41.8	69.1
AP_PISA	21.8	41.8	67.3
CP_BT	20.0	40.0	60.0
CP_TB	16.4	40.0	63.6

CP_MJ2h	14.6	40.0	63.6
CP_Qp	12.7	40.0	67.3
CP_TS	9.1	40.0	67.3
CP_HLPL	18.2	38.2	69.1
CP_BFKV	18.2	36.4	61.8
CP_TD	9.1	36.4	54.6
CONSRANK_val	32.7	34.6	41.8
CP_MJ3h	12.7	34.6	58.2
DDG_V	16.4	32.7	60.0

Table S6. Success rate on the **Score_set** for all the 20 best performing classifiers/scoring functions in terms of percentage of targets featuring at least one correct solution within the top-10 ranked positions. Values for the top-1 and top-100 positions are also reported.

Score_set

**Method**	**Top-1**	**Top-10**	**Top-100**
**TF-hAIkal**	26.7	66.7	66.7
AP_PISA	40.0	53.3	66.7
**RF-hAIkal**	33.3	53.3	60.0
**TF-core**	20.0	53.3	66.7
**PRC-core**	40.0	46.7	53.3
**RF-core**	33.3	46.7	53.3
CP_TB	26.7	46.7	80.0
CP_BT	13.3	46.7	60.0
CP_TSC	13.3	46.7	73.3
CP_D1	13.3	46.7	66.7
**PRC-hAIkal**	26.7	40.0	53.3
CONSRANK_val	26.7	40.0	46.7
SIPPER	6.7	40.0	66.7
CP_BFKV	6.7	40.0	66.7
CP_TEl	6.7	40.0	66.7

DDG_V	13.3	33.3	53.3
PYDOCK_TOT	13.3	33.3	46.7
AP_dDFIRE	6.7	33.3	60.0
PROPNSTS	6.7	33.3	73.3
AP_DFIRE2	13.3	26.7	66.7

Table S8. Weights and importance of features selected from the Final optimization step. The table shows the feature importance for the RFC-haikal classifiers; the average weights of the neurons form the only layer of the PRC-hAIkal classifier, and the average weights from the input layer from the TF-haikal classifier. Depending on the classifier, features have different influences. The most important feature is different for each classifier, while we see a trend on the lower end of values. For example, on the RF, the added features PRC-core and TF-core have the lowest importance; similarly, on TF, they have the lowest negative values alongside CONSRANK_val. PRC-haikal has a different composition of weights where CONSRANK_val is the one with the most negative value, and PRC-core and TF-core have higher scores putting them in the middle of the list. The only consensus from these classifiers is that PRC-core and TF-core contribute about the same amount to the final prediction.

	RFC_hAIkal	PRC_hAIkal	TF2_hAIkal
CONSRANK_val	0.14005727	-29.451714	-1.3212645
CP_HLPL	0.17810914	-3.2093374	-0.6273031
CP_MJ3h	0.0734944	0.12833599	0.31232381
DDG_V	0.16473502	0.68846896	-0.2429195
CP_RMFCA	0.19207643	-0.532189	-0.4458839
AP_GOAP_DF	0.17126157	1.75106146	-0.0955708
PRC-core	0.0366392	-2.3267881	-2.0085049
TF-core	0.04362697	-2.7415448	-1.2761909

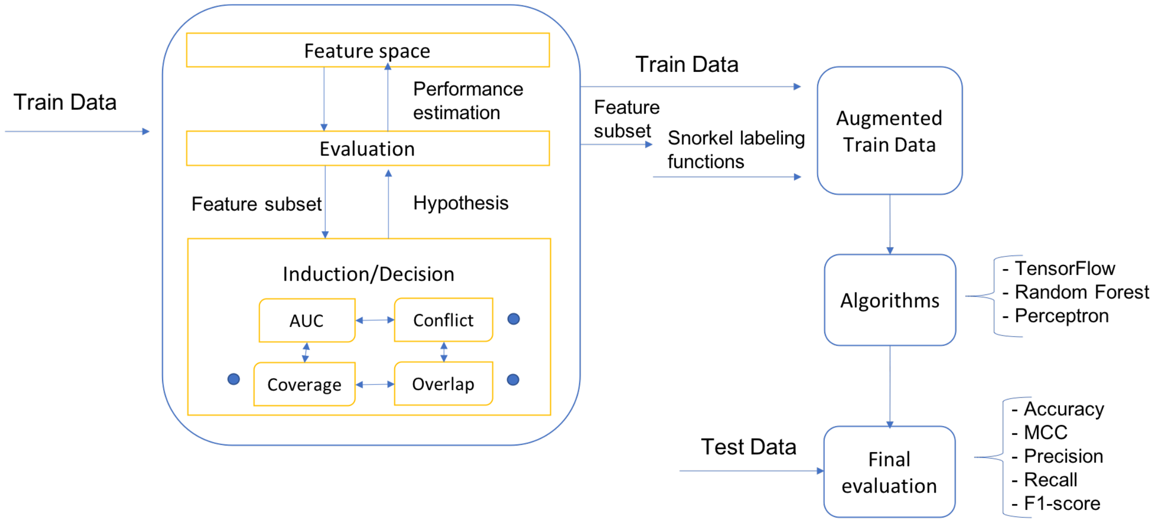

Fig. S6: Feature selection process. From out train date , we wrap the feature selection process, in different steps. starting from all the features available, we evaluated this features using the AUC value and the snorkel built-in evaluation metrics overlap and conflict. We selected the best features to train different algorithms based on high AUC, low conflict , low overlap and certain coverage. The next step was to obtain the final evaluation with our selected features and metrics.

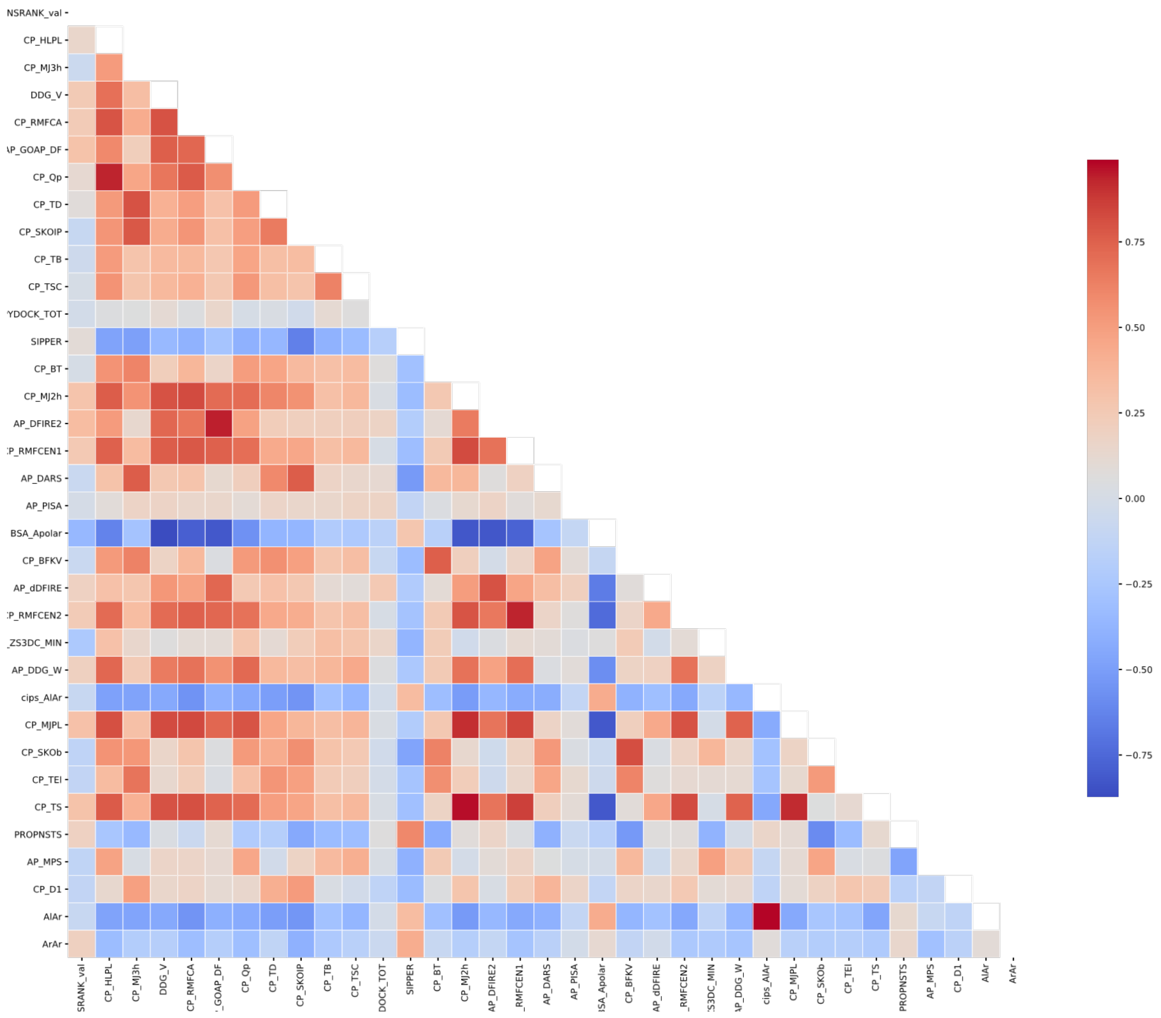

Fig. S7: Pearson correlation of top performing scoring functions according to the ROC on the balanced training data. The linear correlation of the scoring function is indicative of the usefulness to separate the data. The values are in blue-white read color scale, where the lowest correlation have the colorblue, and the highest color red
